# Dataset for random uniform distributions of 2D circles and 3D spheres

**DOI:** 10.1016/j.dib.2022.108318

**Published:** 2022-05-28

**Authors:** Marek Wojciechowski

**Affiliations:** Łódź University of Technology; Faculty of Civil Engineering, Architecture and Environmental Engineering, Al. Politechniki 6, Łódź 90-924, Poland

**Keywords:** Random ditribution of circles, Random distribution of spheres, Homogenization, Representative volume element, Statistical volume element

## Abstract

This dataset contains random uniform distributions for a large number of 2D and 3D balls, along with the description files. It provides the possibility for fast pick up of random, but repeatable, sets of smaller samples, with the guaranteed statistical properties such as random uniform distribution of balls, the predefined expected volume ratio of balls, and also the minimum distance between them. Samples are uniquely identified by the position coordinates in the provided large kernels. The sets of samples can be used in performing numerical predictions of different types for uniform ball distributions while keeping the numerical effort at a reasonable level. Specifically, this can be useful in computational homogenization of fiber and spherical particle reinforced composites, where the provided kernels can be viewed as representative volumes and the samples as the realizations of statistical volume elements. Some secondary results, like the numbers of samples of a given size assuring the required accuracy in expected ball volume ratio representation, are also provided. Data was created by means of the pseudo-random number generator using python scripting and can be loaded and used also in other programming environments.

## Specifications Table

**Table:** 

Subject	Engineering
Specific subject area	Generation of randomly distributed balls, homogenization of fiber reinforced and spherical particle reinforced random composites.
Type of data	Output binary files (.npy format)
	Description text files (.json format)
How data were acquired	Data was aquired by numerical computations with the help of python scripting.
Data format	Raw
	Analyzed
Description of data collection	The dataset contains random uniform distributions for a large number of 2D and 3D balls. The following assumptions were made for generating these distributions: ball diameter is unitary, distribution is random uniform, the expected volume ratio of balls varies from 0.025 to 0.48 for 2D case and from 0.01 to 0.3 for 3D case with the step 0.005, and distance between balls is guaranteed to be greater than a value varying from 0 to 0.05 with the step 0.005. Ball positions for all geometrical configurations were generated with pseudo-random number generator available in NumPy and spatial subpackage present in SciPy.
Data source location	Łódź University of Technology, Łódź, Poland, EU
Data accessibility	Mendeley Data, https://doi.org/10.17632/nbtp99bd76.1
Related research article	M. Wojciechowski, On generalized boundary conditions for mesoscopic volumes in computational homogenization, Composite Structures, 2022, https://doi.org/10.1016/j.compstruct.2022.115718

## Value of the Data

• Dataset is useful because it provides kernels of precomputed positions of 2D and 3D ball centers with guaranteed random uniform distribution and with assumed volume ratio and distances between them. Dataset is important because it can be used for standardized, repeatable, and verifiable computations performed on sets of samples taken from the provided kernels.
• The data can benefit researchers and engineers dealing with random materials such as fiber reinforced composites and spherical particle reinforced composites. Another use is also possible wherever the random uniform distribution of points with guaranteed distance is required.
• Data is used for fast generation of sets of samples of randomly uniformly distributed 2D and 3D balls. These samples are picked up from the provided large kernels of ball centers with known statistical properties (kernels). The benefit is that the sampling procedure is fast and the results are reliable.

## 1 Data Description

Main data is contained in two files “kernels2D.npy” and “kernels3D.npy”, which are the arrays of centers of balls with unitary radius r. Data is stored in NumPy binary format. The shape of these arrays is $\left(n_{c},n_{d},N,D\right)$, where $n_{c}$ is the number of considered ball volume ratios, $n_{d}$ - number of considered distances, N - maximum number of generated centers, and D - space dimension. The “kernels2D.json” and “kernels3D.json” files are the description files stored in JavaScript Object Notation format. They can be previewed in Firefox web browser, edited in any text editor and loaded as dictionary-like data structure in Python, Matlab and other. The most important entries in these files are:

• c - list of considered expected volume ratios c of balls (of length $n_{c}$);
• d - list of considered additional distances d between balls (of length $n_{d}$); the final distance is guaranteed to be not less than d;
• Ncd - array of numbers of ball centers generated for all (c,d) pairs; number of centers is always greater than 100,000;
• nsamples - a dictionary containing data for computing numbers of samples of given size required to obtain a mean value of the ball volume ratio at the required accuracy in relation to the expected volume ratio; entries of this dictionary are:
  – N - list of considered expected numbers of balls N in the sample;
  – R - array of the corresponding radii R of the samples computed for all considered c and N pairs;
  – sigma - estimations of the standard errors in ball volume ratio representation in the set of samples of given size; computed for all c, d, N triplets;
  – nsamples - required numbers of samples for all c, d, N triplets;
  – rmargin - required relative accuracy for which nsamples were generated;
  – confidence - level of confidence for which nsamples were generated;
  this entry should be considered as secondary data.

The data repository contains also “example2D” and “example3D” directories, however, they are not considered as the part of this present data article.

**Fig. 1:**
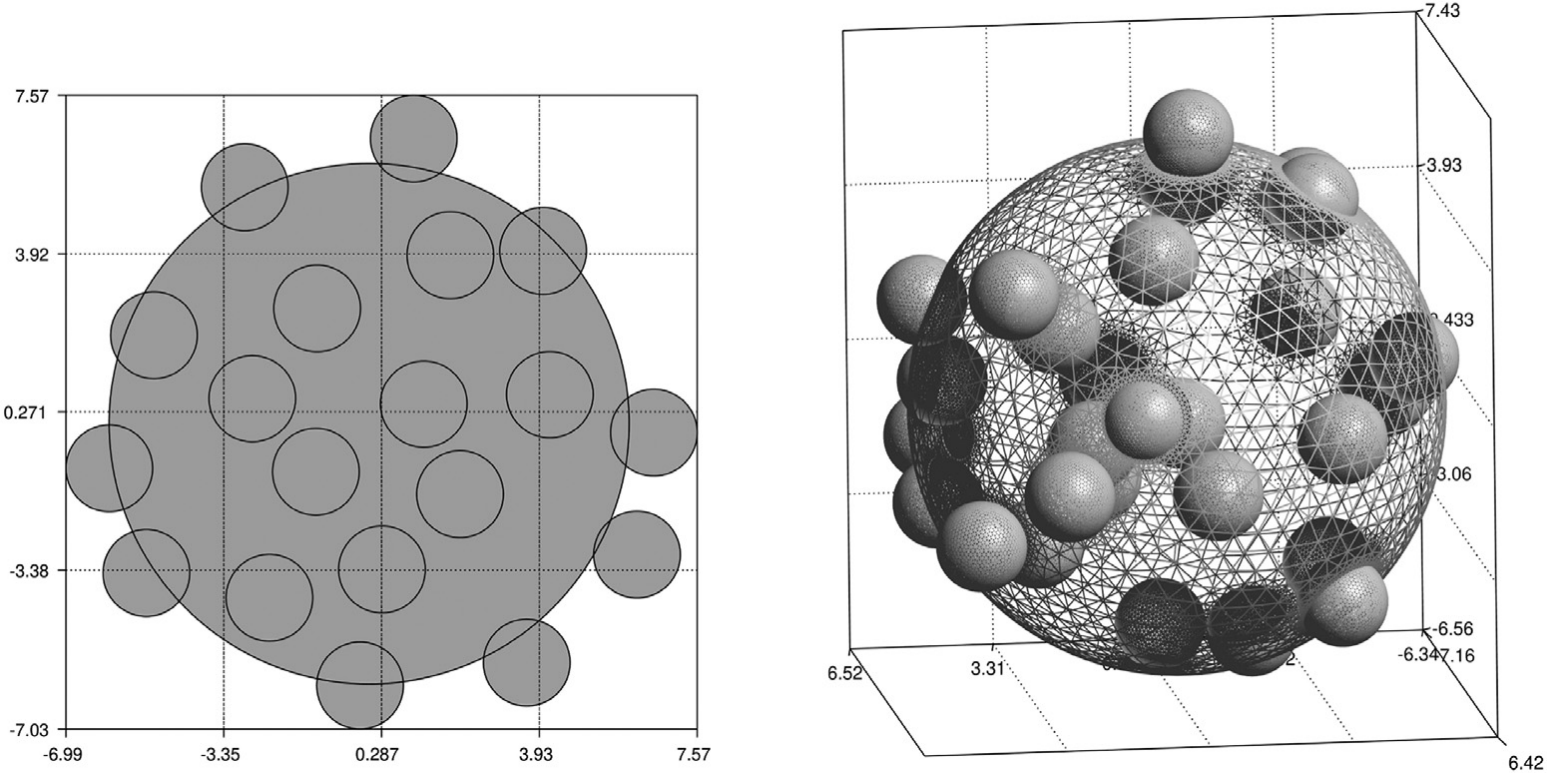
Exemplary samples of balls. On the left: sample taken from “kernels2D.npy” file for c=0.35, d=0.025, r=1, R=6 and P=(10,10). On the right: sample taken from ”kernels3D.npy” file for c=0.065, d=0.025, r=1, R=6 and P=(10,10,10). Volume ratio of the balls contained inside the samples is close to the expected values of c (but not identical).

**Fig. 2:**
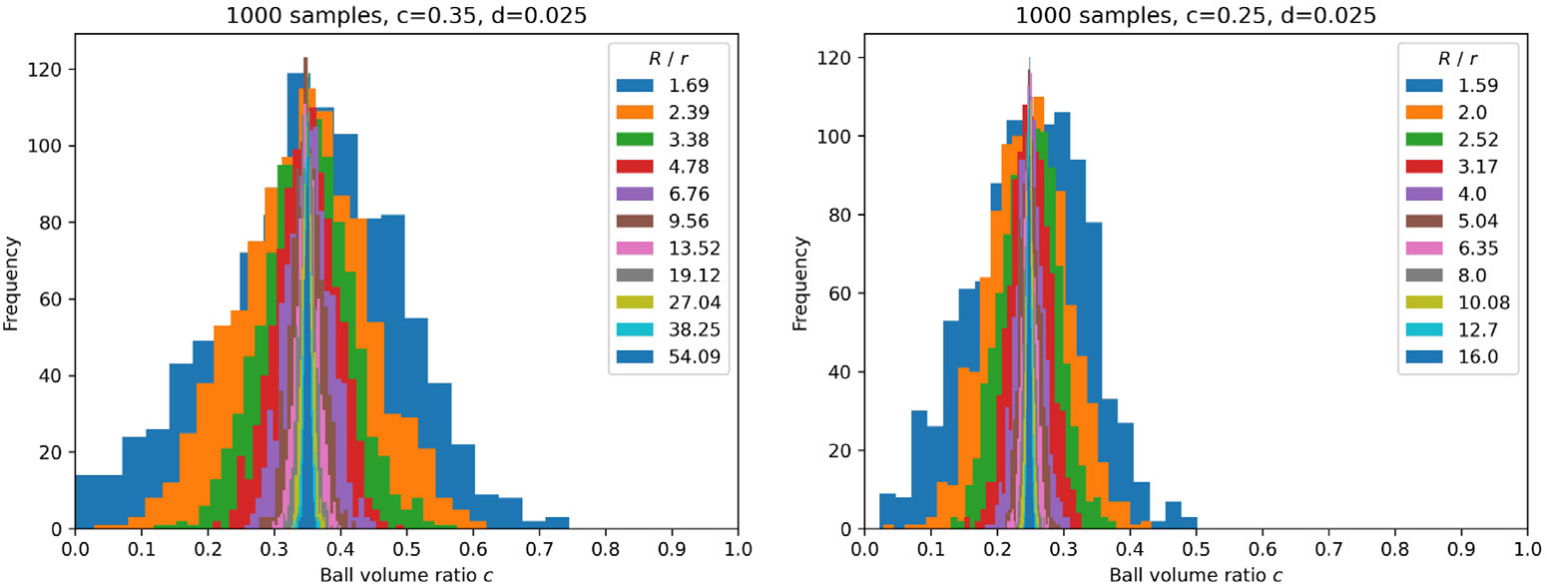
Examples of distributions of ball volume ratio for random sets of samples of different sizes. On the left 2D case (‘kernels2D.npy’), on the right 3D case (‘kernels3D.npy’). Mean values oscillate close to the expected c values and the deviations decrease with sample size.

In order to use this dataset the number of dimensions, ball volume ratio, and the required minimum distance between balls have to be chosen first. Next, the indices i, j in c and d lists for the chosen values have to be determined, respectively. The exact number of centers generated for chosen geometric configuration is then retrieved from Ncd array and finally, the kernel is acquired from appropriate.npy file. Consider this python code snippet as an example:


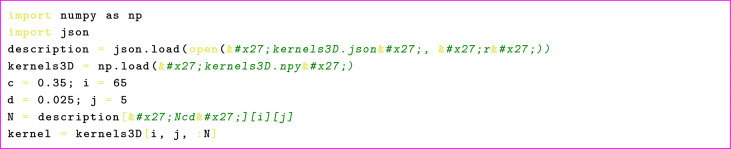
An analogous script will be written for the 2D case. Sampling from the loaded kernel is then performed as follows:


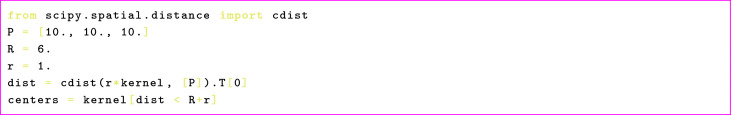
The centers variable contains positions of balls of radius r=1 which intersect the sampling ball of the radius R=6 with the center located at the point P=(10,10,10) in the kernel coordinate system (see Fig. 1). Many randomly (or deterministically) placed samples of a given size can be retrieved from kernels giving the sets of samples of the required statistical properties. See Fig. 2 for the examples of ball volume ratio distributions for large sets of samples of different sizes.

## 2 Experimental Design, Materials and Methods

### 2.1 General setup

The following assumptions were made for generating kernels of ball centers:

• diameter r of balls is unitary;
• distribution of balls is random uniform;
• expected volume ratio of balls c varies from 0.025 to 0.48 for 2D case and from 0.01 to 0.3 for 3D case, with the step 0.005 for both cases;
• distance between balls is guaranteed to be greater than d, a value varying from 0 to 0.05 with the step 0.005.

Lower bounds for the expected volume ratio of balls c are dictated by practical reasons (very sparse ball distributions are rarely needed), whereas the upper bounds are determined by the physical limits rather, as reported in Zubko and Zubko [1]. The minimum distance between balls is also considered for practical reasons, i.e. d>0 is often required when creating high quality finite element meshes on the samples of balls.

Computations were performed using python scripting with the help of NumPy and SciPy packages. Ball centers were sampled using the default random number generator present in the NumPy package (version 1.20.1) with the default settings – see NumPy documentation and also [2] for further reference. The seed number for this generator was left unspecified, i.e. it has been taken automatically from the machine environment at the time of performing computations. Data was generated on the server equipped with two Intel Xeon Gold 5220R CPUs.

### 2.2 Kernel generation details

For ball radius r=1 and for every considered expected volume ratio of the balls c and additional distance between them d, the following procedure was performed:

1. Expected number of balls is assumed as N=100,000.
2. Radius of the spherical domain occupied by the balls is calculated as:
  (1) $$R=r{\left(\frac{N}{c}\right)}^{\frac{1}{D}}.$$
3. List of ball centers $P_{nk}$, where $n=1\cdots \bar{N}$ and k=1⋯D, is initialized with $\bar{N}=0$.
4. Direction vector $\pi_{k}$ of the new ball center is drawn from Gaussian distribution (μ=0, σ=1) and then normalized; this assures that all the generated direction points are distributed in a random uniform way on the surface of the domain with unitary radius [3].
5. Length scale factor ρ for the direction vector $\pi_{k}$ is established using the inverse of a cumulative distribution function (CDF) that corresponds to the surface area of the spherical domain in *D* dimensions, i.e. $\rho =x^{\frac{1}{D}}$ is taken, where x is a number drawn uniformly from the range [0,1].
6. Final position of the ball center is computed as:
  (2) $$p_{k}=\left(R+r\right)\rho \pi_{k}.$$
7. Distances between the newly generated center and other centers added previously to the array $P_{nk}$ is computed as follows:
  (3) $$d_{n}=\sqrt{\sum_{k}{\left(P_{nk}-p_{k}\right)}^{2}}.$$
8. If $d_{n}>\left(2+d\right)r$ for all n, then new point $p_{k}$ is appended to the array $P_{nk}$ (so that $\bar{N}$ increases by 1), otherwise new point is not appended.
9. Steps 4–8 are repeated up to achieving the required ball volume ratio c. Note that the balls can be placed in such a way, that they cross the boundary of the domain and only partially contribute to the overall ball volume. This is true if ((R+r)ρ∈[R−r,R+r]. This possibility is allowed for assuring a truly random uniform distribution of balls inside the domain. The side consequence is that the final number of generated centers is higher, then the expected number of balls, i.e.: $\bar{N}\geq N$.

The arrays $P_{nk}$ generated with the above procedure for space dimensions D=2 and D=3 and for all considered c, d pairs are collected into two arrays and saved in “kernels2D.npy” and “kernels3D.npy” files, respectively, along with the JSON description files. Note, that for obtaining kernels of the identical properties, but for r≠1, it is sufficient to scale the existing coordinates by factor r.

### 2.3 Determination of representative number of samples

Let us assume that the set of samples with radius R<R randomly picked up from the given geometrical configuration (i.e. for given D, c, d) must be such, that the mean volume ratio of balls for this set does not differ from the expected c value by more than the predefined error margin δc with the confidence level A. Using the central limit theorem, it is assumed that the distribution of volume ratio means computed for many sets of samples of a given size tends to the normal distribution with mean value $\mu_{c}=c$. The standard deviation $\sigma_{c}$ depends on radius R (see Fig. 2). The number of necessary samples can be then determined by the following equation:

(4) $$N_{\mathrm{samp}\mathrm{les}}={\left(\frac{Z\left(A\right)\cdot \sigma_{c}\left(R\right)}{\delta c}\right)}^{2},$$

where Z(A) is half of the confidence interval for the normal distribution function. Standard deviations $\sigma_{c}\left(R\right)$ have been estimated for some chosen R values. This is done simply by generating large number of random sets of samples. This number is taken somewhat arbitrarily as $\max\left[250,{\left(\frac{R}{R}\right)}^{D}\right]$. Computed values are placed in sigma variable of nsamples entry in description JSON files, along with the corresponding R values. Using the computed $\sigma_{c}\left(R\right)$ also $N_{samples}$ have been computed for specific choices of confidence parameters, namely:

• δ=0.05,A=0.99→Z(A)=2.579 for 2D case;
• δ=0.05,A=0.95→Z(A)=1.96 for 3D case.

The pre-computed numbers are placed in nsamples variable of nsamples entry of the description JSON files.

## CRediT authorship contribution statement

**Marek Wojciechowski:** Conceptualization, Methodology, Software, Data curation, Writing – original draft, Visualization, Investigation, Validation.

## Declaration of Competing Interest

The author declare that he has no known competing financial interests or personal relationships which have, or could be perceived to have, influenced the work reported in this article.

## Data Availability

Dataset for Random Uniform Distributions of 2D Circles and 3D Spheres (Mendeley Data).
